# Acromegaly Presenting With Cricoarytenoid Joint Arthropathy

**DOI:** 10.1002/lary.70369

**Published:** 2026-01-15

**Authors:** Samantha Salvi Cruz, Riley Larkin, Ioan Lina, Alexander Gelbard

**Affiliations:** ^1^ Department of Otolaryngology – Head & Neck Surgery Vanderbilt University Medical Center Nashville Tennessee USA; ^2^ Department of Otolaryngology – Head & Neck Surgery University of Colorado Anschutz Medical Campus Aurora Colorado USA

**Keywords:** acromegaly, cricoarytenoid, glottic stenosis, subglottic stenosis

## Abstract

Excess growth hormone in acromegaly induces characteristic acral and soft tissue overgrowth (particularly in the face and hands), arthropathies, as well as cardiovascular and metabolic complications. Similar proliferative changes can occur in the larynx, where hypertrophy of the arytenoid and cricoid cartilages may impair vocal fold mobility. We describe a rare case of acromegaly affecting the cricoarytenoid joints (CAJ), leading to proximal airway obstruction and demonstrating that these diarthrodial joints are susceptible to systemic arthropathies.

## Graphical abstract

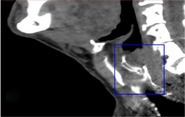

## 1 Introduction

Acromegaly is a rare endocrine disorder caused by excess circulating growth hormone (GH) and insulin‐like growth factor 1 (IGF1), most commonly from a GH‐secreting pituitary adenoma. Patients exhibit characteristic acral and soft tissue overgrowth (particularly in the face and hands), arthropathies, as well as cardiovascular and metabolic complications [1]. Treatment is aimed at normalizing GH and/or IGF1 levels to ameliorate signs and symptoms of the disease and reduce excess mortality. Management of acromegaly as well as the comorbidities created by the disorder is complex and requires a multidisciplinary team of expert physicians. Articular manifestations are a primary cause of morbidity and a major contributor to patient‐reported impairments in quality of life despite long‐term disease remission [2]. The associated arthropathy can affect both axial and peripheral joints, and it may be present as the earliest clinical symptom of the disease.

The human larynx is composed of two mobile vocal cords tethered between the thyroid and arytenoid cartilages. The diminutive arytenoids articulate on the signet‐ring shaped cricoid cartilage in the cricoarytenoid joint (CAJ), a diarthrodial joint supported by a synovia‐lined capsule [3]. The CAJ controls adduction (closing), abduction (opening), and lengthening of the vocal cords, which is essential for respiration, airway protection, and voicing (phonation).

## 2 Case Report

**FIGURE 1:**
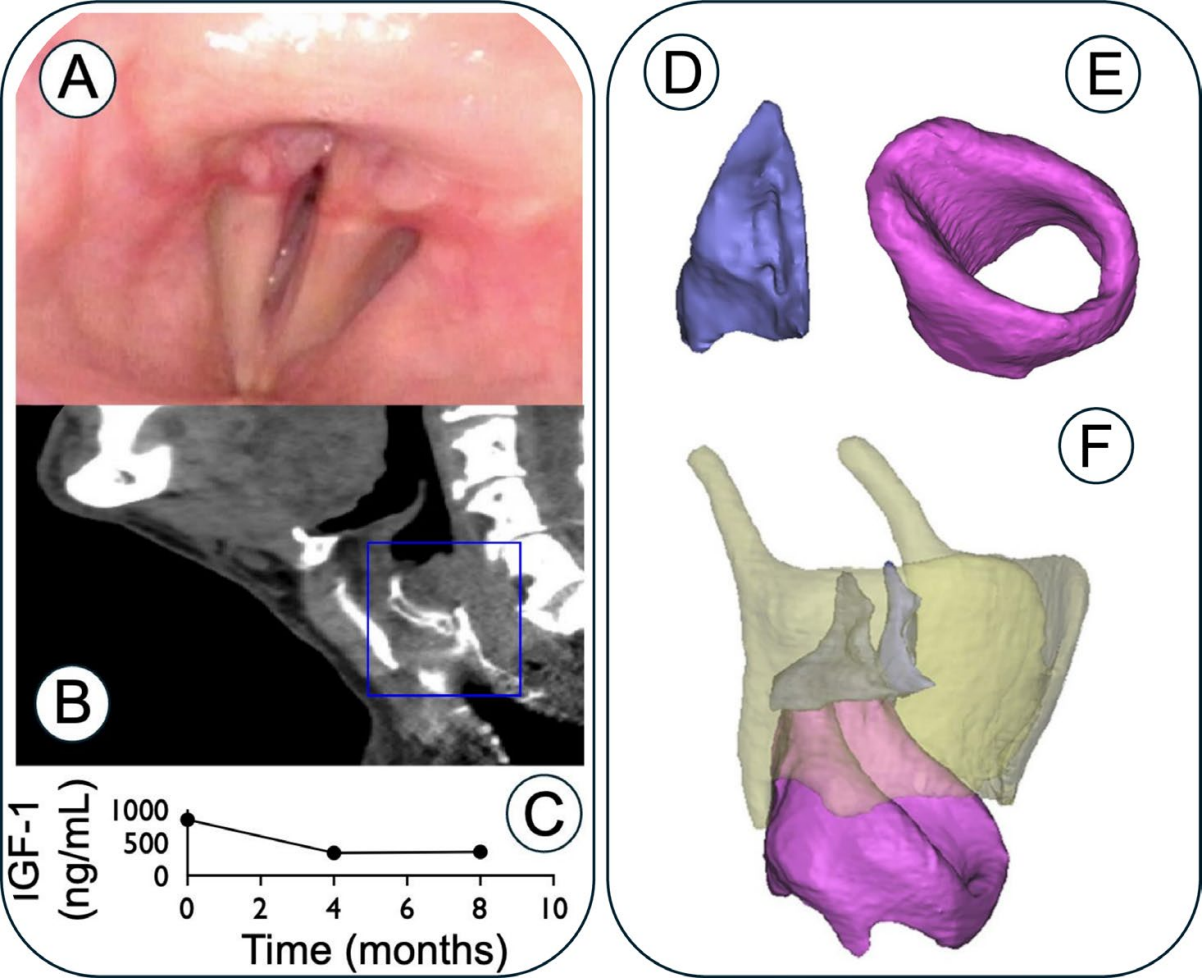
(A) Flexible laryngoscopy at follow‐up. (B) Sagittal CT scan of the neck with area of interest (blue square) highlighting the laryngeal region. (C) Timeline of IGF‐1 levels following diagnosis and initiation of treatment. (D) arytenoid, (E) cricoid, and (F) laryngeal cartilages and their composite framework. *Source*: D, E, and F reproduced with permission from *Journal of Voice*, Vol. 26, No. 5, 2012.

A 72‐year‐old man without a medical history notable for prior surgeries or intubations presented to the laryngology clinic with 6 months of progressive shortness of breath on exertion. On exam, the patient appeared comfortable at rest and could speak in complete sentences. With exertion, he was noted to have audible inspiratory stridor. He demonstrated a subtle prominence of his supraorbital ridge, along with a broad nose and prognathism. His height was above average (6 ft, 5 in.), and he had enlarged hands. Flexible fiberoptic laryngoscopy demonstrated restricted mobility of the bilateral vocal folds (*although they were not immobile*), severe cricoarytenoid joint hypertrophy, and a narrowed glottic airway. Subsequent laboratory investigation demonstrated a growth hormone (GH) level of 23.1 ng/mL (elevated; normal: ≤ 3.0 ng/mL). Adrenocorticotropic hormone (ACTH) and cortisol levels were within normal limits at 17 pg/mL (normal: < 47 pg/mL) and 7.3 mcg/dL (normal: 3.7–19.4 mcg/dL), respectively. IGF‐1 levels were also elevated; for detailed values over time, refer to Figure 1C. A noncontrast computed tomogram (CT) of the neck confirmed hypertrophy of the cricoarytenoid joint complex with elongated arytenoid cartilages and a thickened joint capsule. The remainder of the proximal airway cartilaginous superstructure (trachea and mainstem bronchi) showed no deformity or endoluminal obstruction.

Given the patient's stability at rest, an endocrinology consultation facilitated diagnostic testing confirming a growth hormone‐secreting pituitary adenoma. He initiated treatment with monthly Lanreotide injections (120 mg, *a synthetic form of somatostatin*). Within a month of presentation, he experienced an excellent biochemical response (Figure 1C).

The patient was diagnosed with CAJ arthropathy from a GH secreting pituitary adenoma. His initial dyspnea symptoms related to limited CAJ mobility resulting in a narrow glottic airway. Despite an excellent biochemical response, the CAJ arthropathy did not resolve. Fortunately, the airway remained stable at one year follow‐up. While surgical interventions aimed at static glottic enlargement (to improve airflow and limit subjective dyspnea on exertion) or bypassing the laryngeal obstruction with a tracheostomy were discussed, the patient elected to maintain serial observation given the stability of his symptoms and his ability to maintain an adequate quality of life.

## 3 Discussion

Glottic obstruction results in progressive dyspnea and ultimately respiratory failure if left untreated. Timely diagnosis is therefore essential to prevent complications, preserve quality of life, and improve clinical outcomes. The most common etiologies of glottic obstruction include laryngeal malignancy, fibrotic CAJ contracture from prolonged endotracheal intubation, autoimmune conditions (e.g., granulomatosis with polyangiitis), and infectious diseases (e.g., syphilis, fungal infections, and diphtheria). This case illustrates that CAJ arthropathy can be the presenting sign of acromegaly.

Acromegaly due to excess growth hormone has been reported to result in structural changes in the upper airway, including soft tissue hypertrophy and thickening of the laryngeal and tracheal cartilage [4]. GH and IGF‐1 stimulate the growth of articular cartilage and periarticular ligaments. The most common acromegalic arthropathies involve the large peripheral joints (shoulder, knee, and hip) [5]. These effects, however, are not limited to the appendicular skeleton. In the larynx, hypertrophy of cartilaginous structures can similarly lead to joint hypomobility and glottic narrowing. These laryngeal cartilaginous changes mirror the joint manifestations seen in classical acromegalic arthropathy, where excessive cartilage proliferation results in decreased joint mobility [5]. Collaboration with endocrinology colleagues is critical to address both the local airway effects and systemic hormonal imbalance driving them. Unfortunately, the structural joint changes that occur with excess secretion of GH are frequently not reversible despite successful biochemical control.

## 4 Conclusion

Our patient presented with progressive dyspnea secondary to cricoarytenoid joint arthropathy and glottic obstruction as a consequence of acromegaly. The combination of radiographic findings and impaired vocal fold mobility highlights how acromegaly can drive joint morbidity—even in the larynx. This case illustrates the importance of multidisciplinary care in the management of acromegaly and highlights the nature of the cricoarytenoid joint, which renders it susceptible to arthropathies.

## Funding

The authors have nothing to report.

## Conflicts of Interest

The authors declare no conflicts of interest.

## Data Availability

Data sharing not applicable to this article as no datasets were generated or analyzed during the current study.
